# Physicochemical Properties and Pollen Profile of Oak Honeydew and Evergreen Oak Honeydew Honeys from Spain: A Comparative Study

**DOI:** 10.3390/foods8040126

**Published:** 2019-04-17

**Authors:** María Carmen Seijo, Olga Escuredo, María Shantal Rodríguez-Flores

**Affiliations:** Department of Vegetal Biology and Soil Sciences, University of Vigo, Faculty of Sciences, 32004 Ourense, Spain; mcoello@uvigo.es (M.C.S.); mariasharodriguez@uvigo.es (M.S.R.-F.)

**Keywords:** honey, *Quercus pyrenaica*, *Quercus ilex*, botanical characteristics, physicochemical properties, geographical origin, cluster analysis

## Abstract

This work investigates the similarities and differences of oak honeydew (*Quercus pyrenaica* Willd.) and evergreen honeydew (*Quercus ilex* L.) honey produced in Spain. For this purpose, the physicochemical characteristics of 17 samples from oak honeydew and 11 samples from evergreen honeydew collected in different geographical regions were analyzed. All the samples accomplished European Union requirements for honey consumption. Both honey types had amber dark color; however, the evergreen oak honey was clearer than oak honey, having higher mean values in a* and b* coordinates of CIELab scale. In general, both honey types exhibited high electrical conductivity, a moderate value of pH, medium to low water content, and high diastase activity. The reducing sugar content was significantly lower and maltose content was significantly higher in evergreen honeydew. In addition, total phenols and total flavonoid contents, the antioxidant activity and the melissopalynological analysis was performed. The oak honeydew honey had a higher abundance of *Castanea*, *Rubus* and *Erica* pollen grains, while the evergreen oak honeydew honey had a higher abundance of *Lavandula*, *Olea europaea* or *Anthyllis cytisoides*. A multivariate analysis using the most representative pollen types and physicochemical components facilitated the differentiation of the honey samples, thus this information can be useful for the honey characterization.

## 1. Introduction

Honey is a food produced directly by the honeybees from the apicultural resources existing near apiaries. For this reason, the characteristics of this natural product are closely linked to the production area [1,2]. For honey elaboration, the honeybees collect the nectar of the flowers, plant secretions or excretions whose production is mediated by sucking insects, such as aphids. Considering these sources, honey is classified as nectar or blossom honey or honeydew honey [3]. Honeydew and nectar honey differ in terms of chemical composition, physical properties, and melissopalynological characteristics [4,5,6,7,8,9]. In Spain, a few years ago, honeydew honey was an undervalued product, with a low commercial value; however, it turned out to be very valuable being one of the most demanded honey by consumers [10]. The increase in demand is attributed to its healthy properties due to high antioxidant capacity and phenolic content [1,2,6,7,11,12]. 

**Figure 1:**
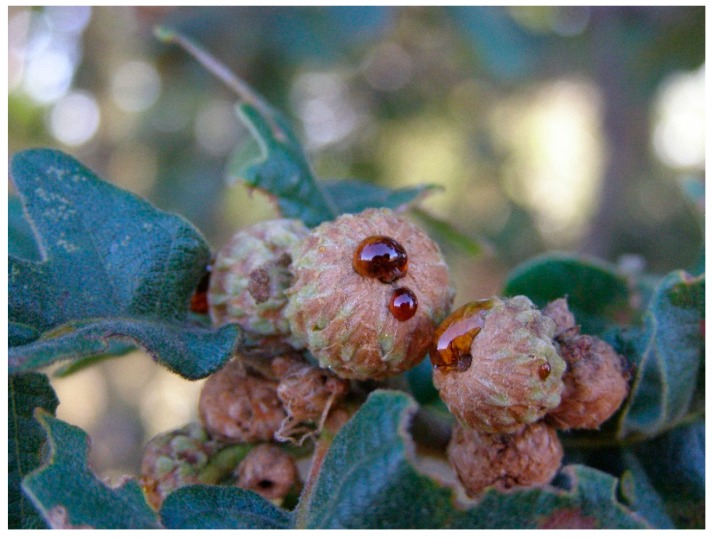
Honeydew produced during the acorn formation of deciduous oaks.

In Europe, honeydew honey is obtained mainly from trees. In the mountain areas, through exudates produced by scale insect, on conifers as *Picea abies*, *Abies*, or pine species [13,14,15,16]. In the Mediterranean areas, using plant secretions produced during the acorn formation in evergreen oaks and deciduous oaks (Figure 1). In the Iberian Peninsula, *Quercus ilex* (the evergreen oak) is biogeographically distributed mainly in central and southern areas, while other species of deciduous oaks, such as *Quercus pyrenaica* appears mainly in the west and north mountain areas [17].

The origin of honeydew, the factors influencing its production and its physicochemical characteristics are insufficiently studied. Honeydew production is not constant throughout the year and depends on the environmental conditions, the physiology and phenology of the plants, and the dynamics of vectors affecting secretions as the biological cycle of insects. In northwest Spain, honeydew honeys are obtained in the summer and their production depends on the weather [1]. Some authors have indicated that rainy springs and warm summers favor the collection of this honey type [18]. Hence, in Mediterranean habitats, honeydew productions are favorable. In recent decades, in the north and northwest regions of the Iberian Peninsula of Mediterranean, the change in climate is causing a significant effect on the phenology of plants and, consequently, in the beekeeping trends. Some of these changes are visible, for example, honeydew is more frequently harvested by beekeepers in the northwest region of the Iberian Peninsula. 

The physicochemical and sensorial characteristics of nectar honey are better known in comparison to those of honeydew honey. In particular, unifloral honeys, such as orange blossom (*Citrus*), eucalyptus (*Eucalyptus*), acacia (*Robinia pseudoacacia*), linden (*Tilia*), lavender (*Lavandula stoechas*), heather (*Erica*, *Calluna vulgaris*), rape (Brassica) or sunflower (*Helianthus annuus*) have been extensively studied [6,8,19,20,21,22,23,24]. The most studied honeydew honeys are those produced in the Mediterranean area and Central Europe. They are produced from oak (*Quercus ilex*), fir (*Picea abies*, *Abies*), or *Pinus* [1,4,5,8,11,15,25]. Honeydew honeys are characterized by dark amber to dark color, slightly intense smell with a predominance of a wood attribute, intense flavor slightly bitter and sour in taste. However, the available information on the sensory characteristics of the honey type is still scarce [26]. These honeys are characterized by less reducing sugar content, higher oligosaccharide content [4,6,27], and higher electrical conductivity, as a consequence of its higher mineral and phenolic content [6,28,29,30]. In addition, the palynological analysis revealed important differences between the honey sediments of honeydew and nectar honeys [4,5,31]. Generally, honeydew honeys harbor more pollen grains from anemophilous plants, fungal hyphae, spores or green algae. At the time of honey classification, the presence or absence of fungal spores and the fungi that give rise to these fungal elements should be considered. This is because some fungal elements are not related to the presence of honeydew in honeys [32]. However, significant relationships between the presence of certain spores of plant pathogenic fungi (e.g., *Alternaria*, *Helminthosporium*, *Uncinula*, etc.) with the honeydew in the honey were found [5]. On the contrary, the presence of yeasts in honey, especially, *Metschnikowia reukafii*, is indicative of the nectariferous origin of honey [5,33,34,35]. 

Several aspects must be considered to guarantee the safety of the food. The improvement in the procedures for the quality control of honey is the first step. The identification of the peculiar characteristics of each honey type, and the differences attributed to the geographical origin or the botanical origin are also of special interest with respect to food safety as they provide information for the traceability of the honey. This knowledge is useful to avoid counterfeit products derived from adulteration or mislabeling of food products. Therefore, the study of palynological and physicochemical characteristics of honeydew honeys produced from deciduous oaks and evergreen oaks collected in different areas of Spain provides crucial information that facilitates their differentiation.

## 2. Material and Methods

### 2.1. Honey Samples

Honeydew honeys were collected from different areas of Spain; 17 samples were collected from the northwest area, and 11 samples from the center-south area (the geographical origin of samples and the collection year is shown as supplementary data). The samples were collected directly by the beekeepers and refrigerated at 4 °C and stored until further analysis. All the determinations were conducted in duplicate, following the homogenization of the samples. 

### 2.2. Reagents and Standards

All chemical standards were HPLC-grade pure. Folin-Ciocalteu reagent, gallic acid, aluminum chloride, sodium carbonate, potassium iodide, and bisulfite were purchased from Panreac (Barcelona, Spain). Quercetin and 2,2-diphenyl-1-picrilhidrazil (DPPH) were purchased from Alfa Aesar (Massachusetts, USA) and methanol was obtained from Merck (Darmstandt, Germany). Hydrolyzed starch for diastase determination was purchased from Carlo Erba (Barcelona, Spain). The calibration of the HANNA Honey Color C221 colorimeter was with glycerin was provided by Glycerol HANNA instruments (Woonsocket, Rhode Island, USA). Standards of glucose, fructose, sucrose, maltose, trehalose, turanose and melezitose were obtained from Sigma–Aldrich (Madrid, Spain).

### 2.3. Melissopalynological Analysis

The botanical characteristics of samples were identified by optical microscopy based on the method established by Louveaux et al. [36], with some modifications. Qualitative analysis was conducted to determine the proportion of pollen grains of different species present, and quantitative analysis was done to determine the amount of pollen per unit weight of honey. Ten grams of honey was dissolved in bi-distilled water to a final volume of approximately 30 mL for qualitative analysis. The samples were subjected to two centrifugations (Sigma Laborzentrifuge centrifuge model 3.0) at 4500 rpm (3383× *g*). The supernatant was decanted, and the sediment was stirred to homogenize it. Subsequently, slides for microscopy were prepared using a drop (100 µL) of sediment. The pollen spectra were determined by counting and identifying a minimum of 800 pollen grains with a Nikon Optiphot II microscope (×400 and ×1000, as and when needed). The proportions of pollen types were expressed as percentages. For quantitative analysis, a volumetric method was used involving mixing 5 g of honey in bi-distilled water. Then, the honey solution was centrifuged as done previously. The sediment was re-dissolved in bi-distilled water until a known volume was obtained. Finally, two aliquots of 10 μL of sediment were used to count the number of pollen grains in them. Results were expressed as the number of pollen grains per gram of honey (pollen grains/g).

### 2.4. Physicochemical Analysis

The moisture of the honey was measured with a digital refractometer (ABBE URA-2WAJ-325; Auxilab S.L., Navarra, Spain). The refractive index values, at 20 °C, were converted to moisture contents using a Chataway table. The pH and electrical conductivity (EC) of a honey solution (5 g honey dissolved in 25 mL bi-distilled water) were measured directly using a pH meter (Crison micro pH 2001; Crison Instruments S.A., Barcelona, Spain) and a portable conductivity meter (Knick Portamess 913 Conductivity, Beuckestr, Berlin), respectively. Finally, the results were expressed as mS/cm. 

The hydroxymethylfurfural (HMF) content and the diastase activity were determined following the methodology proposed by Bogdanov et al. [37]. The HMF content was estimated using the White spectrophotometric method. This method considers the difference between the UV absorbance at 284 nm of a honey solution (0.2 g/mL) and the same solution after adding bisulfite. The HMF level was calculated after subtraction of the background absorbance at 336 nm (Jenway 6305 UV-Visible Spectrophotometer, Staffordshire, UK). Results were expressed in mg/100 g).

The diastase activity was determined on the basis of the hydrolysis rate of the starch solution by the α-amylase present in a honey buffer solution at 40 °C. The method used to determine the diastase activity was the Schade method described by Bogdanov et al. [37]. The amount of starch converted was determined by measuring the absorbance of the honey solution at 660 nm using a UV-VIs spectrophotometer (Jenway 6305 UV-Visible Spectrophotometer, Staffordshire, UK) at different time points until an endpoint when the absorbance was less than 0.235. Diastase activity was calculated as diastase number (DN) or grams of starch hydrolyzed each hour per 100 g honey at 40 °C. 

A HANNA Honey Color C221 colorimeter was used to determine the color in the honey, after calibration with glycerin (Glycerol HANNA instruments). The sample (approximately 4 mL) was placed in a plastic bucket with smooth walls. The honey must be fluid to be analyzed correctly; in the case of crystallized or weakly fluid honey; it is heated up to 45 °C in a thermostatic bath (J. P. SELECTA S.A.) and allowed to stand to eliminate the bubbles. Results were expressed in mm Pfund. The color of the honey was also measured on the CIELab scale. This system defined three colorimetric coordinates (L, a*, and b*), which are dimensionless magnitudes. L coordinate defines the brightness of honey samples; a* and b* are the chromatic coordinates and they represent variation between reddish-green and yellowish-blue, respectively. 

### 2.5. Determination of The Total Polyphenol and Flavonoid Content

For the determination of the phenol content, the Folin-Ciocalteu spectrophotometric method, adapted to honey by Singleton et al. [38] was used. This method was based on the oxidation of the phenolic compounds of phosphomolybdic and phosphotungstic acids, forming a bluish complex, which is analyzed at 765 nm using a UV-Vis spectrophotometer (Jenway 6305, UK). Solutions of honey samples (0.1 g/mL) were prepared. A calibration curve was obtained using gallic acid solutions (0.01–0.50 mg/mL) as a reference standard. The total phenolic content was expressed as gallic acid equivalents in mg/100 g honey.

The total flavonoid content was measured by spectrophotometry using the Dowd method adapted by Arvouet-Grand et al. [39]. The method uses a solution of aluminum chloride that reacts with the flavonoids present in the honey solution (0.33 g/mL). Solutions developed a yellow color for which absorbance was determined spectrophotometrically at 425 nm. The total flavonoid content was determined using a standard curve with quercetin (0.002–0.01 mg/mL) as a reference standard. Finally, the results were expressed as equivalents of quercetin in mg/100 g honey.

### 2.6. Radical Scavenging Activity

The antioxidant activity of honey was determined by the DPPH discoloration method. It involves the determination of the antioxidant capacity of 2,2-diphenyl-1-picrilhidrazil (0.0006 M) by spectrophotometrically [40]. A honey solution in methanol (0.1 g/mL) was prepared. This solution (0.3 m) was mixed with 2.7 mL of a DPPH solution (6 × 10^−5^ M). The sample mixture and blank DPPH solution were maintained in the dark at room temperature for 30 min. Then, the absorbance was measured at 517 nm using a UV-Vis spectrophotometer (Jenway 6305, Staffordshire, UK). Discoloration of the DPPH in each sample was calculated by the percentage of RSA (radical scavenging activity): RSA = [(AbsB−AbsS)/AbsB] × 100, where AbsB is the absorbance of the DPPH solution and AbsS is the absorbance of the honey sample solution. 

### 2.7. Sugar Composition

The sugar composition was determined using an ion Dionex ICS-3000 chromatography system (Sunnyvale, California, EEUU) [6]. The system separated the sugars by using an analytical polyvinylidene/polyvinylbenzene CarboPac PA1 column (Dionex 3 × 250 mm) suitable for mono-, di-, tri-, and oligo-saccharides and a pulsed amperometric detector with a gradient of two mobile phases (A and B). Phase A was ultrapure water, while phase B was 200 mM NaOH (HPLC grade, Merck). The sugars in honey solutions (10 mg/L) were calculated using the calibration curves of the standard solution for each pure sugar. The Chromeleon Chromatography Management System was used for acquisition of the chromatograms. The linear ranges of identified sugars were between 10–45 mg/L (glucose and fructose), 0.5–10 mg/L (sucrose, maltose), 0.1–10 mg/L (turanose, melezitose, and trehalose). The retention time as well the LOD and LOQ are included in the Supplementary Material. Finally, the concentrations of the sugars were expressed as g/100 g honey

### 2.8. Statistical Analysis

The difference between pollen characteristics, physicochemical parameters, bioactive compounds and sugars of both honey types was determined using a Student’s *t*-test. The significance was determined at *p* < 0.05. The cluster multivariate analysis or conglomerate analysis was used as the classification method of honeydew honeys. This statistical approach groups the samples together based on a set of case-variable data. The objective is to place the cases (individuals) in homogeneous groups, suggested by the essence of the data so that individuals that can be considered similar are assigned to the same cluster, while different (dissimilar) individuals are located in different clusters. The cluster analysis allowed classification of honeydew honey samples while considering all the variables (palynological and physicochemical). The statistical analyses were carried out with the SPSS Statistic 23.0 (IBM SPSS Statistics, Armonk, New York, USA) and Statgraphics Centurion 17.0 for Windows (Statgraphics Technologies, Inc., The Plains, VA, USA).

## 3. Results and Discussion

### 3.1. Microscopic Analysis of The Sediment of Honeydew Honeys

#### 3.1.1. Oak Honeydew Honeys

The samples harbored a wide variety of pollen grains. In the oak samples, 33 pollen types belonging to 21 plant families were identified. Table 1 shows the percentage of representation (third column) and the frequency classes (P, R, I, A, D) of the pollen grains. It shows the most frequent pollen types are those found in more than 20% of samples with a frequency in pollen spectra higher than 1%. *Castanea sativa*, *Erica*, *Cytisus* pollen type and *Rubus* pollen were present in all the samples. Other well-represented pollen types were *Echium*, *Frangula alnus*, *Crataegus monogyna*, *Salix*, *Trifolium*, and *Eucalyptus* (>58% of samples). *Sesamoides* and *Frangula alnus* were occasionally found as secondary pollen. The *Quercus* pollen, though present in low levels (less than 3%), is also frequent among these samples (82.4% of samples). The pollen content per gram of honey had a wide range, with an average value of 18451 grains/g.

#### 3.1.2. Evergreen Oak Honeydew Honeys

In the evergreen oak honey samples, 45 pollen types belonging to 24 plant families were identified (Table 2). *Echium* and *Quercus* pollen were present in all samples. Other pollen grains that were well represented in the pollen spectra (more than 80% of representation) were *Brassica*, *Cistus ladanifer* type, *Campanula*, *Cytisus* type, *Lavandula stoechas*, *Rubus*, *Campanula* type, *Taraxacum oficcinale*, *Trifolium* type, *Castanea sativa* and *Eucalyptus*. *Echium*, *Castanea sativa*, and occasionally, *Rubus* and *Anthyllis cytisoides* (in one sample) were found as dominant pollen in some samples. Some pollen types, such as *Galega officinalis*, was found such as important pollen in four samples, *Hypecoum* was identified as dominant pollen in one sample, and *Olea europaea* pollen, present in more than 50% of the samples, was significantly associated with honey sediment. The average pollen content of this group was 38,519 grains/g of honey. Samples had a wide range of variation from 8900 to 149,500 pollen grains/g of honey.

### 3.2. Physicochemical Profile of Honeydew Honeys

The quality and physicochemical characteristics of both groups of honeys are shown in Table 3. 

#### 3.2.1. Oak Honeydew Honeys

The oak honeydew honeys exhibited mean moisture content of 17.4%, mean electrical conductivity of 1.0 mS/cm, mean pH of 4.4, mean HMF content of 0.1 g/100 g, and mean diastase activity of 24.6. They had a dark amber color, with a mean Pfund of 142 mm, moderate values of L coordinate, and low values of a* and b* coordinates. They had high level of phenols (mean value of 134.8 mg/100 g) and flavonoids (mean value of 9.7 mg/100 g) along with a high RSA (mean value of 72.4%).

Another interesting feature when characterizing honey is the content of reducing sugars. The mean content of fructose and glucose was higher than the lower limit for honeydew honeys (45%) and slightly higher than oak honeydew honey (60%), which is the lower limit value indicated in the honey quality standard for blossom honeys. Other quantified sugars were turanose (with a mean value of 3.1%), maltose, sucrose, and melezitose (with values lower than 1%). Among these samples, the trehalose content was not quantified. To predict the degree of crystallization of honey, the ratio between reducing sugars and moisture content were also calculated. The mean values of F/W ratio and G/W ratio were 2.0 and 1.6, respectively.

#### 3.2.2. Evergreen Oak Honeydew Honeys

The evergreen oak honey had a mean moisture content of 16.7%, mean electrical conductivity of 1.1 mS/cm, mean pH of 4.4, mean HMF of 1.3 mg/100 g, and mean diastase content of 27.3. They were dark amber colored honey (120 mm Pfund), with moderate values of L coordinate and slightly elevated values of a* and b* coordinates. The mean phenol and flavonoid content was 111.3 mg/100 g and 7.5 mg/100 g, respectively, and the mean RSA was 63.3%. The mean reducing sugar content in honeydew honeys was less than 60%. The mean turanose and maltose levels were 2.4 g/100 g and 1.2 g/100 g, respectively. Mean melezitose, sucrose, and trehalose levels were 0.9 g/100 g, 0.2 g/100 g, and 0.2 g/100 g, respectively. The G/W ratio was 1.4.

### 3.3. Differentiation Between Both Honeydew Honeys 

**Figure 2:**
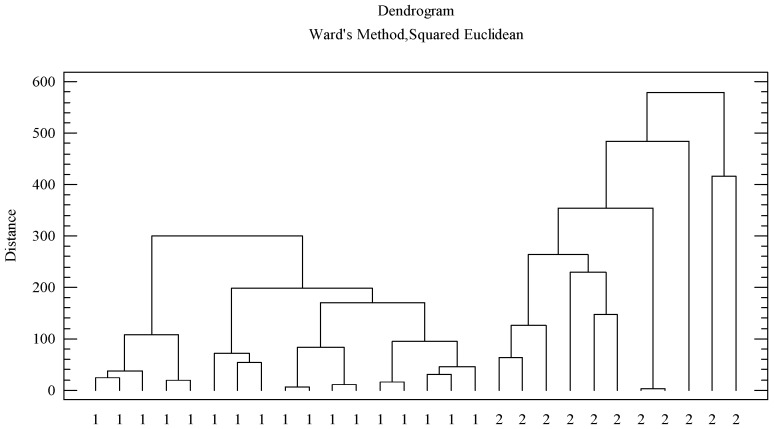
Cluster analysis of the honey samples. 1: oak honeydew. 2: evergreen oak honeydew.

Despite the similarities in both honey types, in physicochemical parameters and in pollen content, multivariate techniques are useful for the classification and differentiation of honey samples. Cluster analysis is a technique of data reduction that facilitates classification of the observations in homogeneous subgroups based on a diverse set of data. This statistical procedure identifies similarities between the different cases grouping them. The data used for this purpose were the physicochemical variables, color, polyphenolic and sugars content, and the variables obtained by the palynological analysis that are better represented in the samples. Pollen types that were found in very low frequency (<1%) or were poorly represented were not taken into account. Thus, honeydew samples were classified into two clearly differentiated subgroups (Figure 2). The first cluster (namely 1) included the oak honeydew honeys (17 samples). While the second cluster (identified with the number 2) included the evergreen honeydew honeys (11 samples). As could be observed, the first group (cluster 1) exhibited the highest similarities between samples, since they had smaller square Euclidean distances compared to the second group (cluster 2). This suggested that samples from evergreen oak exhibited more differences among themselves compared to the samples obtained from deciduous oak. Considering that honey characteristics are associated with the territory from which it is obtained and that the biogeographical area in the Iberian Peninsula occupied by *Quercus ilex* is more diverse than the corresponding area occupied by *Quercus pyrenaica*, the evergreen oak samples are expected to exhibit higher heterogeneity. 

#### 3.3.1. Differences in The Pollen Profile of The Two Groups of Honeydew Honeys

In order to establish statistical differences attributable to the pollen content and diversity of both groups of samples, a *t*-test for independent samples was performed (Table 1 and Table 2). The presence of some pollen types was significantly different between both types of honey. For example, a greater frequency of *Echium* was observed in evergreen oak honey (*p* < 0.01), whereas a greater frequency of *Erica* was observed in oak honey (*p* < 0.01). Significantly higher levels of *Lavandula* pollen were found in evergreen oak honey compared to in oak honeys (*p* < 0.01). While *Frangula alnus* pollen was significantly higher in the oak honey (*p* < 0.01). Other pollen types that collaborated in the differentiation of these honey samples were *Brassica*, *Campanula*, *Cistus ladanifer*, *Carduus*, *Helianhtus annuus*, *Taraxacum officinale*, *Galega officinalis*, *Quercus*, and *Olea europaea*, since they are found in higher percentage in evergreen oak honey (*p* < 0.05). On the contrary, *Cytisus* was significantly higher in oak honeys (*p* < 0.01).

The pollen diversity of the honey constitutes evidence of the honeybee plant resources in the area where honey was obtained, although, factors such as plant biology, honeybee colony dynamics, beekeeping practices, and environmental conditions affect the pollen diversity and the pollen content of honey. The pollen spectrum of a honey sample showed the history of the product and even their geographical production environment is particularly important for the traceability of the product. The most significant differences between both groups of samples correspond to the presence or higher abundance of taxa related to the Mediterranean vegetation in evergreen oak. This is the case of *Lavandula*, *Olea europaea*, *Anthyllis cytisoides*, *Galega officinalis*, *Ceratonia siliqua* and *Hypecoum*, or pollens of crop plants more common in this area, such as *Helianthus annuus* or *Olea europaea*. On the contrary, in oak honey, the pollen of species such as *Castanea*, *Rubus*, and genus *Erica* is better represented. Although Spain produces honeydew honey, there have been few studies on its botanical characterization. Similar results with respect to the dominant pollen were found by Escuredo et al. [5] and Rodríguez-Flores et al. [1], due to the same geographical origin of samples. Terrab et al. [10] analysed honeydew honeys produced in diverse provinces of the country, demonstrating that a pollen combination of *Quercus*, *Rubus ulmifolius*, *Campanula erinus*, *Capsella*, *Cytisus scoparius*, *Echium plantagineum*, *Cistus ladanifer*, and *Castanea sativa* is characteristic of Spanish oak honeydew honeys. However, while analyzing a specific geographical origin (province) of honeydew honeys, the influence of the Mediterranean vegetation against the Atlantic vegetation (northern Spain) is more appreciated [10], as is shown in the present study.

#### 3.3.2. Differences in the Physicochemical Profile of the Two Groups of Honeydew Honeys

Both honey types had different colors. Oak honeydew was darker than evergreen honeydew (*p* < 0.01). The a* and b* coordinates were higher in evergreen oak honeydew honeys compared to oak honeydew honeys (*p* < 0.01). In addition, significantly higher phenol content (*p* < 0.05), higher flavonoid content, and higher RSA content (*p* < 0.01) were found in oak honeydew honeys. The oak honeydew honeys showed significantly lower content of reducing sugars (*p* < 0.05), and in the sum of both sugars (*p* < 0.01). On the other hand, the maltose content was slightly higher for the evergreen oak honeydew group (*p* < 0.05). Finally, the G/W ratio of evergreen oak honeydew honeys was significantly lower (*p* < 0.05). 

The analysis of physicochemical characteristics revealed the influence of the origin territory on the production of honey [20]. The honey samples collected from the central and southern areas of Spain, with a drier climate, exhibited lower moisture content. Both groups of honeydew honeys complied with current legislation in terms of freshness parameters, as revealed by the HMF content lower than 40 mg/kg and values of diastase activity greater than 8 [3]. All the honey samples exhibited an electrical conductivity higher than 0.800 mS/cm. These values are consistent with those published by other authors for honeydew honey obtained from different areas of origins [1,4,20,41,42]. It is also noteworthy the nectariferous secretions of *Erica* plants may contribute to higher phenolic content and higher sugar content (especially, glucose) in oak honeydew honeys since according to some authors, honey obtained from northwest Spain exhibits these qualities [6,24]. 

The differentiation between honeydew honey samples using only phenolic compounds is a complicated task. Simova et al. [7] distinguished the oak honey from other honeydew honeys, such as fir and spruce, using spectroscopic techniques. Some researchers demonstrated the possibility of distinguishing the honeydew honey of *Quercus* sp., evergreen oak, and Hungarian oak from other as Silver fir and conifer samples, due to the difference in profiles with respect to phenolic content, antioxidant activity, and certain quality parameters [43]. Antioxidant compounds in honey samples produced in different climates are a marker of floral origin and a potential indicator of their biological quality [44]. Hence, there is a close relationship between some predominant flora and the antioxidant activity of honey obtained from northwest Spain, due to the high presence of per example, *Erica* or *Castanea* [23,24]. On the other hand, some studies have reported that honeys identified as dark amber-colored honey (such as oak honeydew honeys or chestnut honeys) has a higher antioxidant capacity, deriving from their phenolic compositions [7,9,45], which enhances its beneficial effects on human health due to its high antioxidant and antimicrobial activity [2,43,46]. The darkness in the color predominated in all the studied honeydew honeys; however, redness and yellowness were higher in the samples obtained from evergreen oak honeydew. Honey color depends on the flora involved and on associated pigment, polyphenols, flavonoids, and mineral content [6,9]. The influence of its botanical and geographical origin is reflected in the close relationship found between the color coordinates a* and b* with the most representative pollen types of honeys analyzed. *Lavandula stoechas* type, *Echium*, *Helianthus annuus* type, *Taraxacum oficcinale*, *Carduus* type, and *Cistus ladanifer* type positively correlated with a* and b* coordinates of CIELab (R^2^ > 0.40; *p* < 0.05). While, *Castanea sativa*, *Cytisus*, *Rubus*, and *E. cinerea* negatively correlated (R^2^ > 0.40; *p* < 0.05) with the coordinates. This could explain the small nuances that influence the color of both groups of honeydew honeys.

The honeydew honey samples usually have a reducing sugar content lower than 60%, however, evergreen oak honeydew honeys exhibited lower reducing sugar content. Several authors have shown that honeydew honey has a lower content of reducing sugars [4,15,27,45], while others have indicated similar values to those obtained for oak honeydew honeys [1,9,22]. The sugar content and water levels are related to the crystallization phenomenon of honey. Honeys with low glucose content (<30%) undergo a slower granulation process over time [47]. When the G/W ratio is under 1.7, a slowly crystallizing honey is produced [48,49]. The F/G and G/W ratios are also useful for the classification of unifloral honeys [4,22,47,48], in the case of honeydew honeys that are reported to remain liquid for the longest [50,51]. With respect to minor sugars, some trisaccharides are also used as markers of honeydew in honeys, especially, melezitose [20,49,52,53], erlose, or raffinose [50,54]. In general, the honeydew honeys obtained from Spain had a lower content in these minor sugars compared to the honeydew honey obtained from Central European countries [46,49,52]. Pascual-Maté et al. [50] reported that the mean concentration of these trisaccharides in Spanish oak honeydew honeys was higher than the corresponding mean values found in our study. Rybak-Chmielewska et al. [15] reported that the European fir honeydew honeys exhibited a higher mean melezitose content than oak honeydew samples. Although the main component of honey is sugar, the use of these chemical compounds to differentiate honeydew honey samples is complex.

## 4. Conclusions

Honeydew honey samples obtained from different botanical origins and biogeographical areas of production were studied to analyze the similarities and differences between them. Despite, certain similarities in some physicochemical parameters, such as the dark amber color, high electric conductivity, medium-low water content, and high polyphenol content, some differences were observed between both honey types. The more relevant differences included higher a* and b* coordinates of the color measured in the CIElab scale in evergreen oak honeydew. The oak honeydew honey had higher polyphenol and flavonoid content, which was probably related to the higher antioxidant activity of this group of samples. Although the melissopalynology is not useful to identify the botanical origin of this type of honey, it provides useful information about the geographical origin of the honey samples and could be used as a tool to follow the traceability of the product. 

## Figures and Tables

**Table 1:** Percentage of representation and frequency classes of the main pollen types in oak honeydew honeys.

Family	Pollen type	%	P	R	I	A	D
Ericaceae	Erica **	100.0	5.9	47.1	41.2	5.9	-
Fabaceae	*Cytisus* type	100.0	-	17.6	82.4	-	-
Fagaceae	*Castanea sativa*	100.0	-	-	-	64.7	35.3
Rosaceae	*Rubus*	100.0	-	-	-	58.8	41.2
Fagaceae	*Quercus*	82.4	76.5	5.9	-	-	-
Boraginaceae	Echium *	76.5	52.9	11.8	11.8	-	-
Caprifoliaceae	Frangula alnus *	64.7	47.1	5.9	5.9	5.9	-
Rosaceae	*Crataegus monogyna* type *	64.7	41.2	17.6	5.9	-	-
Salicaceae	*Salix*	64.7	64.7	-	-	-	-
Fabaceae	*Trifolium* type	58.8	52.9	5.9	-	-	-
Myrtaceae	*Eucalyptus*	58.8	23.5	29.4	5.9	-	-
Plantaginaceae	*Plantago*	52.9	52.9	-	-	-	-
Brassicaceae	*Brassica* type	47.1	47.1	-	-	-	-
Campanulaceae	*Campanula* type	41.2	41.2	-	-	-	-
Cistaceae	*Cistus psilosepalus*	41.2	35.3	5.9	-	-	-
Poaceae	Poaceae *	41.2	41.2	-	-	-	-
Poaceae	*Zea mays*	35.3	35.3	-	-	-	-
Resedaceae	*Sesamoides*	35.3	23.5	5.9	-	5.9	-
Rosaceae	*Prunus* type	35.3	35.3	-	-	-	-
Umbelliferae	*Conium maculatum* type	35.3	35.3	-	-	-	-
Labiatae	Lavandula stoechas **	29.4	29.4	-	-	-	-
Compositae	*Centaurea*	23.5	23.5	-	-	-	-
Fabaceae	*Lotus* type	23.5	23.5	-	-	-	-
^1^ Pollen grain/g: 18451 ± 12895							

Letters indicated different frequency classes in the pollen spectra of the samples. P: present pollen (<1%). R: minor pollen (1%–3%). I: important pollen (3%–15%). A: secondary pollen (15%–45%). D: dominant pollen (>45%). %: percentage of representation. * significant differences between the honey type by Fisher’s least significant difference (*p* < 0.05). ** significant differences between the honey type by Fisher’s least significant difference (*p* < 0.01). ^1^ Mean value ± standard deviation.

**Table 2:** Percentage of representation and frequency classes of the main pollen types in evergreen oak honeydew honeys.

Family	Pollen type	%	P	R	I	A	D
Boraginaceae	Echium **	100.0	9.1	18.2	27.3	27.3	18.2
Fagaceae	Quercus *	100.0	45.5	27.3	27.3	-	-
Brassicaceae	*Brassica* type *	90.9	36.4	45.5	9.1	-	-
Cistaceae	*Cistus ladanifer* type *	90.9	54.5	27.3	9.1	-	-
Fabaceae	*Cytisus* type **	90.9	27.3	36.4	27.3	-	-
Labiatae	*Lavandula stoechas* type **	90.9	63.6	27.3	-	-	-
Rosaceae	Rubus **	90.9	9.1	-	36.4	36.4	9.1
Campanulaceae	*Campanula* type *	81.8	36.4	27.3	18.2	-	-
Compositae	Taraxacum oficcinale *	81.8	72.7	9.1	-	-	-
Fabaceae	*Trifolium* type	81.8	54.5	18.2	9.1	-	-
Fagaceae	*Castanea sativa*	81.8	9.1	-	18.2	27.3	27.3
Myrtaceae	*Eucalyptus*	81.8	45.5	27.3	9.1	-	-
Rosaceae	*Prunus* type	72.7	45.5	18.2	9.1	-	-
Umbelliferae	*Conium maculatum* type	72.7	63.6	9.1	-	-	-
Compositae	*Carduus* type *	63.6	63.6	-	-	-	-
Ericaceae	Erica *	63.6	18.2	45.5	-	-	-
Poaceae	Poaceae **	63.6	63.6	-	-	-	-
Salicaceae	*Salix*	63.6	63.6	-	-	-	-
Oleaceae	Olea europaea *	54.5	27.3	27.3	-	-	-
Plantaginaceae	*Plantago*	45.5	27.3	18.2	-	-	-
Brassicaceae	*Diplotaxis*	36.4	27.3	9.1	-	-	-
Chenopodiaceae	*Chenopodium*	36.4	36.4	-	-	-	-
Cistaceae	*Cistus salviifolius*	36.4	36.4	-	-	-	-
Cistaceae	*Halimium* type	36.4	36.4	-	-	-	-
Compositae	*Anthemis*	36.4	27.3	-	9.1	-	-
Compositae	*Helianthus annuus* type *	36.4	27.3	9.1	-	-	-
Fabaceae	Galega officinalis *	36.4	-	-	36.4	-	-
Fabaceae	*Vicia* type	36.4	36.4	-	-	-	-
Labiatae	*Mentha* type	36.4	36.4	-	-	-	-
Fabaceae	*Anthyllis cytisoides*	27.3	9.1	9.1	-	-	9.1
Fabaceae	*Ceratonia*	27.3	18.2	9.1	-	-	-
Fabaceae	*Onobrychis*	27.3	18.2	9.1	-	-	-
Lythraceae	*Lythrum*	27.3	27.3	-	-	-	-
Papaveraceae	*Hypecoum*	27.3	18.2	-	9.1	-	-
Scrophulariaceae	*Scrophularia* type	27.3	27.3	-	-	-	-
Caprifoliaceae	*Frangula alnus*	18.2	9.1	9.1	-	-	-
^1^ Pollen grain/g: 38519 ± 41187							

Letters indicated different frequency classes in the pollen spectra of the samples. P: present pollen (<1%). R: minor pollen (1%–3%). I: important pollen (3%–15%). A: secondary pollen (15%–45%). D: dominant pollen (>45%). %: percentage of representation. * significant differences between the honey type by Fisher’s least significant difference (*p* < 0.05). ** significant differences between the honey type by Fisher’s least significant difference (*p* < 0.01). ^1^ Mean value ± standard deviation.

**Table 3:** Multiple-Sample comparison between both honey types by Fisher’s least significant difference (LSD).

	Mean ± SD	
	Oak Honeydew	Evergreen Oak Honeydew
Physicochemical parameters		
Moisture (%)	17.4 ± 1.0	16.7 ± 1.0
EC (mS/cm)	1.0 ± 0.1	1.1 ± 0.2
pH	4.4 ± 0.2	4.4 ± 0.2
HMF (mg/100 g)	0.1 ± 0.1	0.3 ± 0.6
DI	24.6 ± 4.2	27.3 ± 8.3
Colour (mm Pfund)	142 ± 11 **	120 ± 19 **
L	52.1 ± 3.4	53.8 ± 3.4
a *	7.2 ± 1.5 **	13.9 ± 1.6 **
b *	4.8 ± 1.9 **	14.7 ± 4.8**
Antioxidant properties		
Phenols (mg/100 g)	134.8 ± 26.7 *	111.3 ± 26.0 *
Flavonoids (mg/100 g)	9.7 ± 1.8 **	7.5 ± 1.3 **
RSA (%)	72.4 ± 6.84 **	63.3 ± 8.9 **
Sugar composition (g/100 g)		
Fructose	35.4 ± 1.8 *	32.0 ± 3.9 *
Glucose	27.2 ±1.3 **	24.1 ±1.7 **
Turanose	3.1 ± 0.8	2.4 ± 1.1
Maltose	0.7 ± 0.4 *	1.2 ± 1.0 *
Sucrose	0.2 ± 0.1	0.2 ± 0.1
Melezitose	0.1 ± 0.1	0.9 ± 2.2
Trehalose	nd	0.1 ± 0.1
Fructose + Glucose	62.5 ± 2.4 **	56.2 ± 4.8 **
Ratio sugar/water content		
F+G/W	3.6 ± 0.2	3.4 ± 0.4
F/W	2.0 ± 0.2	1.9 ± 0.3
G/W	1.6 ± 0.1 *	1.4 ± 0.1 *

EC: electrical conductivity; F: fructose; G: glucose; W: water; nd: not detected. * Significant statistical differences at *p* < 0.05. ** Significant statistical differences at *p* < 0.01.

## Supplementary Materials

The following are available online at https://www.mdpi.com/2304-8158/8/4/126/s1, Table S1: Detailed information on the location and date of each honey sample; Table S2: Retention time, linear range, LOD and LOQ for each sugar identified.
