# Supplementary material for: Physicochemical Properties and Pollen Profile of Oak Honeydew and Evergreen Oak Honeydew Honeys from Spain: A Comparative Study
Source: Foods. 2019 Apr 17;8(4):126. doi: 10.3390/foods8040126 (PMC6518035; doi:10.3390/foods8040126)
Supplement: Supplementary file 1 [file foods-08-00126-s001.pdf]

Supplementary material:

Table 1. Detailed information on the location and date of each honey sample.

Sample N.	Type	Year	Locality	Province
1	1	2016	Sober	Lugo
2	1	2017	Sober	Lugo
3	1	2016	Trives	Ourense
4	1	2016	A Limia	Ourense
5	1	2017	Verín	Ourense
6	1	2016	Verín	Ourense
7	1	2017	Verín	Ourense
8	1	2016	Viana do Bolo	Ourense
9	1	2017	Celanova	Ourense
10	1	2016	Verín	Ourense
11	1	2017	A Limia	Ourense
12	1	2016	Viana do Bolo	Ourense
13	1	2017	Viana do Bolo	Ourense
14	1	2016	A Gudiña	Ourense
15	1	2016	Sanabria	Zamora
16	1	2017	Sanabria	Zamora
17	1	2016	Sanabria	Zamora
18	2	2016	Ciudad Rodrigo	Salamanca
19	2	2016	Alba de Tormes	Salamanca
20	2	2016	Guijuelo	Salamanca
21	2	2016	Torrequemada	Cáceres
22	2	2017	Almadén	Ciudad Real
23	2	2016	Monfrague	Cáceres
24	2	2017	Monfrague	Cáceres
25	2	2016	Malpartida	Cáceres
26	2	2017	Badajoz	Badajoz
27	2	2016	La Garrovilla	Badajoz
28	2	2016	Hellín	Albacete

Table 2. Retention time, linear range, LOD and LOQ for each sugar identified.

Sugar	Rt (min)	Linear range (mg/L)	LOD (mg/L)	LOQ (mg/L)
Fructose	14.3	10-45	0.73	2.42
Glucose	12.5	10-45	0.67	2.23
Sucrose	17.6	0.5-5	0.13	0.44
Maltose	38.3	0.5-10	0.28	0.93
Melezitose	25.7	0.15-4	0.02	0.07
Trehalose	3.7	0.25-10	0.11	0.36

Rt.: Retention time; LOD: Limit of detection and LOQ: Limit of quantification
